# Elytra reduction may affect the evolution of beetle hind wings

**DOI:** 10.1007/s00435-017-0388-1

**Published:** 2017-11-18

**Authors:** Jakub Goczał, Robert Rossa, Adam Tofilski

**Affiliations:** 1 Institute of Forest Ecosystem Protection, Faculty of Forestry, University of Agriculture in Krakow, 29 Listopada 46, 31-425, Krakow, Poland; 2 Department of Pomology and Apiculture, Faculty of Biotechnology and Horticulture, University of Agriculture in Krakow, 29 Listopada 54, 31-425 Krakow, Poland

**Keywords:** Beetle, Elytra, Evolution, Wings, Homoplasy, Brachelytry

## Abstract

Beetles are one of the largest and most diverse groups of animals in the world. Conversion of forewings into hardened shields is perceived as a key adaptation that has greatly supported the evolutionary success of this taxa. Beetle elytra play an essential role: they minimize the influence of unfavorable external factors and protect insects against predators. Therefore, it is particularly interesting why some beetles have reduced their shields. This rare phenomenon is called brachelytry and its evolution and implications remain largely unexplored. In this paper, we focused on rare group of brachelytrous beetles with exposed hind wings. We have investigated whether the elytra loss in different beetle taxa is accompanied with the hind wing shape modification, and whether these changes are similar among unrelated beetle taxa. We found that hind wings shape differ markedly between related brachelytrous and macroelytrous beetles. Moreover, we revealed that modifications of hind wings have followed similar patterns and resulted in homoplasy in this trait among some unrelated groups of wing-exposed brachelytrous beetles. Our results suggest that elytra reduction may affect the evolution of beetle hind wings.

## Introduction

The Coleoptera order encompasses almost the quarter of all currently known animal species (Grimaldi and Engel 2005; Hunt et al. 2007). Due to unique evolutionary adaptations, beetles were able to successfully colonize most terrestrial as well as water habitats. Conversion of forewings into hardened shields called elytra is considered to be the adaptation that has played major role in the evolutionary success of beetles (Crowson 1981; Fédrigo and Wray 2010; Grimaldi and Engel 2005; Lawrence 1982). Despite this fact, the molecular basis and the evolution of beetles’ elytra remains largely unexplored (Fédrigo and Wray 2010; Tomoyasu et al. 2009). Since the Hox gene was identified as a key factor in the halteres formation in Drosophila flies (Carroll et al. 1995; Weatherbee et al. 1998), it was believed that the same mechanism determines wing modification in all other insects, including beetles. However, recent studies have provided evidence that formation of elytra in beetles is less affected by Hox gene than previously expected (Tomoyasu et al. 2009).

There is some empirical evidence that elytra protect beetles against predators, support desiccation tolerance, minimize the effect of rapid temperature shifts and protect the hind wings against damage (Linz et al. 2016). Moreover, it was shown that elytra may play a key role in mimicry (Bezzerides et al. 2007) and camouflage (Wilts et al. 2012). In the face of these benefits that arise from the presence of elytra, it is interesting why some beetles have reduced their shields. This rare evolutionary phenomenon is called brachelytry (Jolivet 2005) and its origin, implications and adaptive significance remains unclear.

In general, three different forms of brachelytry may be found in beetles. In the first form, elytra reduction occurs along with the reduction of hind wings. This phenomenon occurs in particular, in females of several beetle species belonging to the families Drilidae, Omalisidae, Lycidae, Lampyridae (Bocák et al. 2013; Bocák and Bocáková 2006; Bocak and Brlik 2008), Vesperidae, the subfamily Prionine (Cerambycidae), subfamily Cebrioninae (Elateridae), and in some species of subfamily Galerucinae (Chrysomelidae) (Jolivet 2005). In the second form of brachelytry, elytra are truncated but completely cover the folded hind wings, which remain functional. This form of brachelytry is the most common. It can be found in particular among Staphylinidae, Silphidae, and Histeridae (Jolivet 2008). The third form of brachelytry is much rarer, and encompasses the species with reduced elytra and exposed (or partially exposed) functional hind wings. Such beetles most often form small distinct taxa among the families of predominantly macroelytrous beetles, e.g., genus *Molorchus* (Cerambycidae) or genus *Malthinus* (Cantharidae). Brachelytrous beetles with exposed hind wings may also be found in the family Ripiphoridae, subfamily Necydaline (Cerambycidae) and subfamily Atractocerinae (Lymexylidae). The elytra reduction varies greatly, irrespective of the brachelytry form, from slightly truncated in Histeridae to the almost completely reduced elytra in Atractocerinae. Apart from the family Staphylinidae, where brachelytry is widespread and even suggested to be the autaphomorphic character (Naomi 1985), this phenomenon is rare and occurs with various forms and intensity in unrelated taxa, which suggests that it has evolved independently several times (Beenen and Jolivet 2008).

It is well documented that reduction of hind, flight wings in beetles has resulted in several significant anatomical modifications, in particular reduction of flight muscles and reduction of nerves, changes in metathorax size and shape, and elytral fusion (Jackson 2012; Rüschkamp 1927; Verma et al. 2014). However, in the case of brachelytry, there is a lack of empirical studies intended to investigate the implications of this phenomenon on the evolution of other morphological traits. The most interesting, and otherwise poorly studied form of brachelytry, encompasses those beetles with reduced elytra and exposed hind wings. Examples of this form are dispersed among several unrelated beetle taxa which makes it possible to investigate how parallel elytra loss influenced the hind wings. Selander (1959) presumed that there may be some parallel morphological modification of hind wings in this group. However, to date, this hypothesis has never been tested empirically.

In this paper, we focused on rare group of wing-exposed brachelytrous beetles. We have investigated whether the elytra loss in distinct groups of beetles is accompanied with the hind wing shape modification, and whether these modifications are similar among far related taxa. For this purpose, we compared the hind wing shape between brachelytrous beetles and their macroelytrous relatives. The comparison was conducted independently within seven distinct beetle taxa.

## Materials and methods

### Species studied

In this study, we have used 39 species (24—macroelytrous and 15—wing-exposed brachelytrous species) from six beetle families: Cantharidae, Oedemeridae, Lymexylidae, Cerambycidae, Meloidae and Ripiphoridae (Table 1). Due to lack of comprehensive phylogeny data for most of the studied species, the analysis was based on the seven independent comparisons conducted within distinct taxa—family or subfamily (Table 1). Within each group we had chosen brachelytrous and macrolytrous representatives (Crowson 1955; Pakaluk and Ślipiński 1995). It was assumed that morphological divergence between the members of the same family or subfamily will be lower than the divergence between unrelated taxa. Phylogenetic independence of the seven formed comparisons was based on comprehensive genetic investigations (Bocak et al. 2014; Hunt et al. 2007).

**Table 1:** Species of beetles used for comparison of brachelytry with macroelytry

No.	Brachelytrous	Macroelytrous	Relation
1	*Malthinus flaveolus* (Herbst, 1786) [2]	*Rhagonycha fulva* (Scop., 1763) [9] *Cantharis fusca* L., 1758 [11] *Cantharis rustica* Fallén, 1807 [2]	The same family
	Cantharidae	Cantharidae	
2	*Oedemera femorata* (Scop., 1763) [12]	*Chrysanthia geniculata* Heyden, 1877 [2] *Nacerdes fulvicollis* Scop. 1763 [2] *Ischnomera cinerascens* (Pandellé, 1867) [2] *Calopus serraticornis* (L., 1758) [8]	The same family
	Oedemeridae	Oedemeridae	
3	*Atractocerus brasiliensis* (Lepeletier and Audinet-Serville, 1825) [2]	*Melittomma brasiliense* (Laporte, 1832) [2] *Elateroides dermestoides* (L., 1761) [11] *Lymexylon navale* (L., 1758) [4]	The same family
	Lymexylidae	Lymexylidae	
4	*Molorchus minor* (L, 1758) [13] *Glaphyra umbellatarum* (Schreber 1759) [10] *Molorchus marmottani* Brisout de Barneville, 1863 [2] *Stenopterus kraatzi* Pic, 1892 [2] *Stenopterus rufus* (L., 1767) [1]	*Trichoferus campestris* (Faldermann, 1835) [2] *Hylotrupes bajulus* (L., 1758) [7] *Plagionotus detritus* (L., 1758) [5] *Obrium brunneum* (Fabricius, 1793) [2] *Phymatodes testaceus* (Linnaeus, 1758) [3]	The same subfamily
	Cerambycidae: Cerambycinae	Cerambycidae: Cerambycinae	
5	Necydalis ulmi Chevrolat, 1838 [2] *Necydalis major* L., 1758 [7] *Callisphyris macropus* Newman, 1840 [2]	*Pidonia lurida* (Fabr., 1793) [9] *Pachyta quadrimaculata* (L., 1758) [9] *Rhagium inquisitor* (L., 1758) [5] *Stictoleptura rubra* (L., 1858) [11]	Necydalinae and Lepturinae are closely related taxa (Hunt et al. 2007)
	Cerambycidae: Necydalinae	Cerambycidae: Lepturinae	
6	*Sitaris muralis* (Forster, 1771) [4]	*Epicauta sibirica* (Pallas, 1773) [2] *Lytta vesicatori*a (L., 1758) [4] *Mylabris quadripunctata* (L., 1767) [2] *Zonitis immaculata* (Olivier, 1789) [2]	The same family
	Meloidae	Meloidae	
7	*Macrosiagon pusilla* (Gerstaecker, 1855) [1] *Metoecus paradoxus* (L., 1761) [3] *Metoecus satanus* (Schilder, 1924) [1]	*Pelecotomoides tokejii* Nomura and Nakane, 1959 [6]	The same family
	Ripiphoridae	Ripiphoridae	

It is believed that the brachelytry evolved independently at least seven times. Each of the origins forms a separate comparison. Numbers in square brackets indicate number of specimens used

### Wing measurements

Both the left and right wing from each specimen were dissected, straightened and mounted between two microscopic slides (Goczał et al. 2016). Subsequently, high resolution (4800 dpi) wing images were obtained using an Epson V330 Photo scanner. Hind wing shape was described by 50 semi-landmarks located on the wing outline (Chazot et al. 2016; Dapporto and Bruschini 2012; Gunz and Mitteroecker 2013) using IdentiFly v. 0.31 software (Przybyłowicz et al. 2015). The first landmark was positioned at the humeral plate. All other semi-landmarks were positioned at equal distances along the wing outline. Semi-landmark configurations were aligned using a sliding semi-landmark method (Bookstein 1997; Mitteroecker and Gunz 2009; Perez et al. 2006; Tocco et al. 2011) with tpsRelw v. 1.54 software (Rohlf 2010). The first and the last landmark were fixed; all others were treated as sliding semi-landmarks.

### Statistical analyses

The hind wing shape was described by ten principal components selected based on the scree plot. The multivariate analysis of variance (MANOVA) was used to investigate the significance of differences in hind wing shape between macro- and brachelytrous beetles in Statistica v. 10 (StatSoft Inc 2011). Procrustes distance (PD) was employed as a measure of morphological divergence. The distances were also used to build a UPGMA similarity tree in the phangorn package (Schliep 2011) in R (R Development Core Team 2015). Subsequently, the principal components of hind wing shape were averaged for brachyelytrous and macroelytrous in each of seven comparisons independently and the differences were visualized using MorphoJ v. 1.06a software (Klingenberg 2011).

## Results

**Fig. 1:**
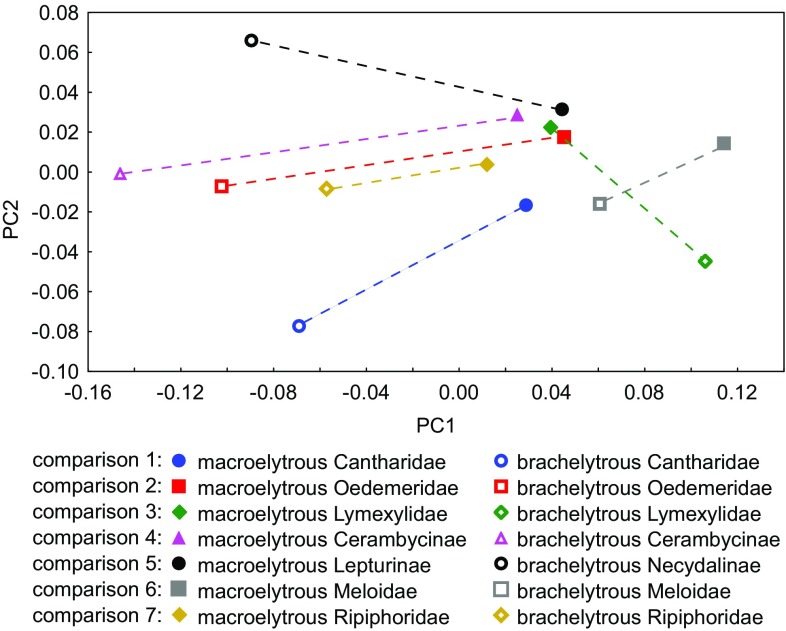
Variation of hind wing outline described by first two components of principal component analysis. The wing outline was averaged within taxa. Empty markers represent brachelytrous taxa and filled markers represent macroelytrous taxa. Markers representing related taxa are connected with lines

Two distinct clusters of points can be found at the scatter plot of the first two principal components of the hind wing shape (Fig. 1). The first principal component allowed separating most brachelytrous and marcroelytrous beetles. However, two species with shortened elytra, Atractocerus brasiliensis (marked with a green empty diamond), and Sitaris muralis (marked with a gray square) were closer to the macroelytrous species. Although, Sitaris muralis was more similar to macroelytrous beetles, it differed from macroelytrous relatives in similar way as in other comparisons—mainly in the first principal component (Fig. 1).

Hind wing shape differed between macro- and brachelytrous beetles (MANOVA: Wilks’ Lambda = 0.0504; *P* < 0.001) and between the comparisons (MANOVA: Wilks’ Lambda = 0.0169; *P* < 0.001). The interaction between the two factors was also significant (MANOVA: Wilks’ Lambda = 0.0399; *P* < 0.001).

**Fig. 2:**
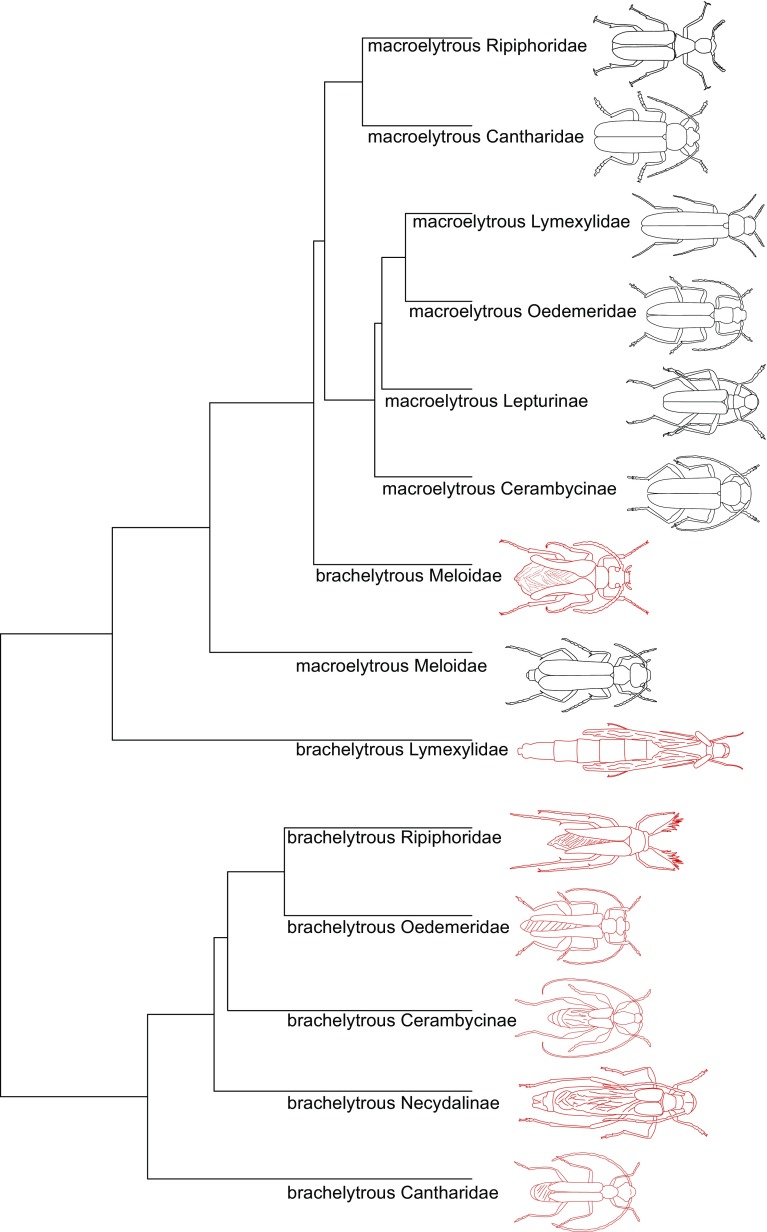
UPGMA similarity tree of hind wing shape of macroelytrous and brachelytrous beetles. Brachelytrous beetles are in red

Macroelytrous beetles formed a separate clad on the UPGMA similarity tree (Fig. 2), whereas most of beetles with reduced elytra were outside of this cluster (Fig. 2). The hind wing shape of brachelytrous Cerambycinae was more similar to the brachelytrous Necydalinae (PD = 0.095) than to macroelytrous species in the same subfamily (PD = 0.176). Oedemerid beetles with shortened elytra were more similar to brachelytrous Cantharidae (PD = 0.083) than to Oedemeridae with normal elytra (PD = 0.151). Brachelytrous Necydalinae were more similar to brachelytrous Cerambycinae (PD = 0.095) and even brachelytrous Oedemeridae (PD = 0.086) rather than to its macroelytrous sister taxa Lapturinae (PD = 0.144). Brachelytrous Ripiphoridae were more similar to the brachelytrous Oedemeridae (PD = 0.064) than to its macroelytrous congeners from the same family (PD = 0.081).

**Fig. 3:**
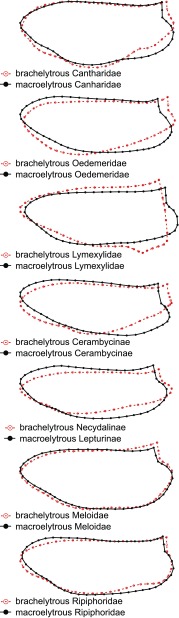
Difference in hind wing shape between macroelytrous and brachelytrous beetles

The pattern of hind wing modification seems to be consistent among some unrelated taxa of brachelytrous beetles (Fig. 3). In brachelytrous Cantharidae, Oedemeridae, Cerambycinae, Necydalinae, Meloidae and Ripiphoridae the reduction occurred with a different intensity along the bottom edge of the anal and marginal fields (Fig. 3). Similar changes are particularly noticeable among Cerambycinae, Necydalinae and Oedemeridae with reduced elytra. In those cases, the hind wings are longer and markedly narrower than in its macroelytrous congeners (Fig. 3). On the other hand, the hind wing of brachelytrous Atractocerus brasiliensis (Lymexylidae) fell out of these patterns. Its hind wing is wider than in macroelytrous congeners and has an elongated anal field (Fig. 3).

## Discussion

We found that hind wings shape differ markedly between brachelytrous and macroelytrous beetles from the same or related subfamily. In most cases, brachelytrous beetles belonging to unrelated taxa were more similar to each other in terms of hind wing shape, than to their macroelytrous relatives. Moreover, our results indicated that in six out of seven comparisons conducted within distinct beetle taxa, the changes in hind wing shape seem to follow the same pattern. The hind wings of brachelytrous and macroelytrous beetles differed mainly in the size and shape of anal and marginal fields (Fig. 3).

The only exception to this pattern was the family Lymexylidae where the wing shape change was different than in other families. We suspect that the exception may be related to the specific flight mode of Atractocerus brasiliensis. It was shown that Atractocerus has a unique flight technique which involves movement of the reduced elytra that play a role similar to that of dipteran halteres (Miller 1971; Taylor and Krapp 2007).

The parallel changes in hind wing shape in wing-exposed brachelytrous beetles have an unclear origin. The evolution of beetles’ hind wings is relatively well studied (Fedorenko 2015; Kukalova-Peck 1978; Kukalová-Peck and Lawrence 2004), however, the role of brachelytry in this process remains unexplored. Because the same physical forces act on different taxonomic groups, changes in wing shape could evolve in parallel among unrelated taxonomic groups. Therefore, it is possible that similar modifications of hind wing shape may arise from optimizing the aerodynamic efficiency or changes in flight mechanics induced by elytra loss. It is known that the occurrence of elytra affects beetle flight mechanics (De Souza and Alexander 1997; Johansson et al. 2012; Le et al. 2013; Sitorus et al. 2010) by increasing lift and reducing aerodynamic efficiency (Johansson et al. 2012). Therefore, it can be expected that the change of flight mechanics will be accompanied by a modification of wing shape. Another explanation of the similar modification of hind wings in wing-exposed brachelytrous beetles may be related to the fact that beetle elytra, despite significant modification, have maintained the genetic identity of wings. It was shown that exclusion of the same wing genes resulted in reduction of both forewings and elytra (Clark-Hachtel et al. 2013). Since these two traits are conjugate to some degree, it is possible that reduction of elytra will entail some modification of hind wings. Another explanation of this phenomenon may arise from the potential mimicry of the studied species. Many Cerambycinae and Necydalinae with reduced elytra are considered to be Hymenoptera mimics (Linsley 1959). Therefore, it is possible that elytra shortening along with the exposure and modification of wings may serve to improve similarity to the model.

The data provided here suggest that there is a relationship between elytra shortening and outline of hind wings. However, nature of this relationship remains unknown. It is not clear if the shorter elytra affect the hind wings directly or there is another factor which affects both size of the elytra and wing outline.

Comparison of insect wings was widely used in phylogenetic investigations (Browne and Scholtz 1995; Comstock 1893; Dijkstra and Kalkman 2012; Kukalová-Peck and Lawrence 2004). However, there are studies which show that phylogenetic information present in insect wings is limited due to processes of reversals, parallel evolution, and convergence (Klingenberg and Gidaszewski 2010). Our results show that in wing-exposed brachelytrous beetles, there was a parallel evolution of the wing outline which can hinder reconstruction of their phylogeny based on wing morphometry alone. In contrast to wing outline, wing venation can be more suitable for reconstruction of phylogeny, thus it is less likely to be under strong selective pressure. The wing venation was found to be suitable for reconstruction of phylogeny in several taxonomic groups (Bai et al. 2012; Perrard et al. 2014; Rossa et al. 2016).

The parallel modification of wing shape was documented in several different groups of insects and may be acquired by various evolutionary mechanisms (Klingenberg and Gidaszewski 2010). Similarity in wing shape may be the effect of adaptation to the specific microhabitat (Chazot et al. 2016), environmental conditions (Pezzoli et al. 1997), migration (Suárez-Tovar and Sarmiento 2016) or be related with an analogous defense strategy (Barber et al. 2015) and behavior (Johansson et al. 2009; Penz and Heine 2016). Here we documented, for the first time, that reduction of elytra can affect hind wing evolution and lead to homoplasy in this trait among unrelated insect taxa.
